# CD8+ TIL Recruitment May Revert the Association of MAGE A3 with Aggressive Features in Thyroid Tumors

**DOI:** 10.1155/2014/921864

**Published:** 2014-11-04

**Authors:** Mariana Bonjiorno Martins, Marjory Alana Marcello, Fernando de Assis Batista, Lucas Leite Cunha, Elaine Cristina Morari, Fernando Augusto Soares, José Vassallo, Laura Sterian Ward

**Affiliations:** ^1^Laboratory of Cancer Molecular Genetics, Faculty of Medical Sciences (FCM), University of Campinas (Unicamp), 126 Tessália Vieira de Camargo Street, Barão Geraldo, 13083-887 Campinas, SP, Brazil; ^2^Department of Biological Sciences and Health, State University of Roraima (UERR), Rua Sete de Setembro 231, 69306-530 Boa Vista, RR, Brazil; ^3^Department of Pathology, AC Camargo Hospital, Antonio Prudente Foundation, 211 Professor Antonio Prudente Street, 01509-010 São Paulo, SP, Brazil; ^4^Laboratory of Investigative and Molecular Pathology, CIPED, Faculty of Medical Sciences (FCM), University of Campinas (Unicamp), 126 Tessália Vieira de Camargo Street, 13083-887 Campinas, SP, Brazil

## Abstract

*Background*. We aimed to investigate a possible role of MAGE A3 and its associations with infiltrated immune cells in thyroid malignancy, analyzing their utility as a diagnostic and prognostic marker. *Materials and Methods*. We studied 195 malignant tissues: 154 PTCs and 41 FTCs; 102 benign tissues: 51 follicular adenomas and 51 goiter and 17 normal thyroid tissues. MAGE A3 and immune cell markers (CD4 and CD8) were evaluated using immunohistochemistry and compared with clinical pathological features. *Results*. The semiquantitative analysis and ACIS III analysis showed similar results. MAGE A3 was expressed in more malignant than in benign lesions (*P* < 0.0001), also helping to discriminate follicular-patterned lesions. It was also higher in tumors in which there was extrathyroidal invasion (*P* = 0.0206) and in patients with stage II disease (*P* = 0.0107). MAGE A3+ tumors were more likely to present CD8+ TIL (*P* = 0.0346), and these tumors were associated with less aggressive features, that is, extrathyroidal invasion and small size. There was a trend of MAGE A3+ CD8+ tumors to evolve free of disease. *Conclusion*. We demonstrated that MAGE A3 and CD8+ TIL infiltration may play an important role in malignant thyroid nodules, presenting an interesting perspective for new researches on DTC immunotherapy.

## 1. Introduction

The largest family of cancer testis antigens (CTAs) and one of the most important is the family of melanoma antigen encoding genes (MAGE). Members of the MAGE family are involved in resistance to apoptosis and cell cycle progression and have been associated with some features of neoplastic phenotype such as immortality, invasion, and immune evasion metastatic ability [1–3]. One of its members, MAGE A3, named melanoma antigen encoding gene A3 because it was first reported to encode a tumor-specific antigen on melanoma cell line [4], has been studied and used in trials of immunotherapy in various histological types of cancers because it is recognized by T-lymphocytes [5, 6].

In tumor cells, these genes are abnormally activated thanks to demethylation of their promoters [7]. Abnormal expression of MAGE A3 gene has been observed in melanomas, lung carcinomas, head and neck cancers, leukemia, squamous cell carcinomas, breast cancers, multiple myelomas, astrocytomas, ovarian tumors, hepatocellular carcinomas, colorectal carcinomas, and thyroid carcinomas, and its abnormal expression is related to aggressive disease in most tumors, a fact that made many authors hypothesize on a possible role of MAGE A3 in malignant transformation and progression [4, 7–9]. Although associated with features of aggressiveness, MAGE A3 might also play an important and beneficial role in tumors, acting as a stimulant of immune responses. In fact, this protein has been tested on several in vivo models, aiming to determine its efficacy as an immune response inducer, and these preliminary results point out to a positive role of MAGE A3 in inducing tumor attack [10, 11]. Although these tests have been conducted in different types of malignancy, studies of MAGE A3 protein in thyroid cancers are scarce and do not relate this protein to immune responses.

The present study aimed to investigate a possible role of MAGE A3 in thyroid malignancy, analyzing its utility as a diagnostic and prognostic marker. We also aimed to analyze whether MAGE A3 expression would be associated with the presence of infiltrated immune cells.

## 2. Materials and Methods

### 2.1. Patients

**Figure 1:**
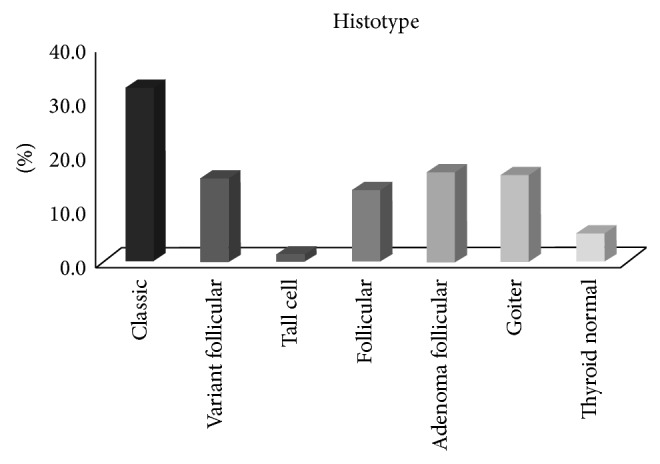
Histological types distributed in TMA.

We studied 314 patients who underwent thyroid resection for thyroid cancer or thyroid nodules at AC Camargo Cancer Center, São Paulo, Brazil. Regarding histological types, 154 out of the 195 malignant tissues were papillary thyroid carcinomas (PTC) and 41 follicular thyroid carcinomas (FTC). The most frequent histotype among papillary carcinomas was the classic variant (CPTC) (101 cases), followed by the follicular variant (FVPTC) (49 cases). The tall-cell (TCPTC) variant was identified in four cases. We also obtained benign tissue from patients, including 51 follicular adenomas (FA) and 51 goiter (G), and 17 normal thyroid tissues obtained from the contralateral lobe of FA (NT) (Figure 1).

Aggressiveness at diagnosis was ascertained using American Joint Committee on Cancer (AJCC) TNM system for differentiated thyroid carcinomas [12]. Patients were managed according to LATS and ATA guidelines [12, 13] and followed for a period of 12 to 175 months (median = 35; mean = 41.1; SD = 26 months).

This study protocol was approved by the Research Ethics Committees of the institutions involved (1259/09-C). All the available clinical, surgical, and pathology reports, as well as follow-up data, were recorded.

### 2.2. Thyroid Specimens

All tumors were carefully and independently reviewed by two experienced pathologists (JV and FAS) for diagnostic confirmation, and cases presenting conflicting results or areas of poor differentiation were excluded. Paraffin blocks of formalin-fixed tissues were collected and, in each case, the most representative area of the tumor, normal surrounding tissue, tumor areas of invasiveness, and metastatic tissue were selected and microdissected whenever available. Tissue microarrays (TMAs) were built using the semiautomated TMArrayer (Beecher Instruments, Silver Springs, MD, USA). Triplicates were obtained from every tissue type, whenever possible.

### 2.3. Immunohistochemical (IHC) Detection of MAGE A3, CD8, and CD4

Five micrometer sections of TMA were placed on electrically charged slides, deparaffinized, and rehydrated in decreasing concentrations of alcohol. The endogenous peroxide activity was blocked with H_2_O_2_ for 15 min. All tissue sections were subjected to heat-induced antigen retrieval using 10% citrate buffer (10 mM, pH 6.0) in a steamer (90°C for 30 minutes). Tissues sections were then incubated overnight at 6°C, with anti-MAGE A3 rabbit polyclonal antibody (LS-B884-50UG, Lifespan Biosciences, Seattle, WA,USA), diluted at 1 : 10; CD4 monoclonal 1F6 (Novocastra, Newcastle upon Tyne, UK) diluted at 1 : 100; CD8 monoclonal SP16 (Thermo Scientific, Rockford, IL, USA) diluted at 1 : 100. The advanced biotin-free polymer detection system was used (DAKO, Carpenteria, CA, USA). DAB (3.3-diaminobenzidine-tetrahydrochloride; Sigma, St Louis, MA, USA) was applied as chromogen for five minutes, at room temperature. Sections were counterstained with hematoxylin. Positive and negative controls were run in the same batch of reaction.

### 2.4. Immunohistochemical Evaluation

Slides were quantified by at least two of the authors (MBM and/or MAM and ECM) and then submitted to other two independent experienced pathologists (JV and FAS), both blinded to tumor features for the final score. An individual evaluation of each marker was completed for each spot tissue, estimating the number of positive cells per TMA spot, considering an approximate area of 0.79 mm^2^.

**Figure 2:**
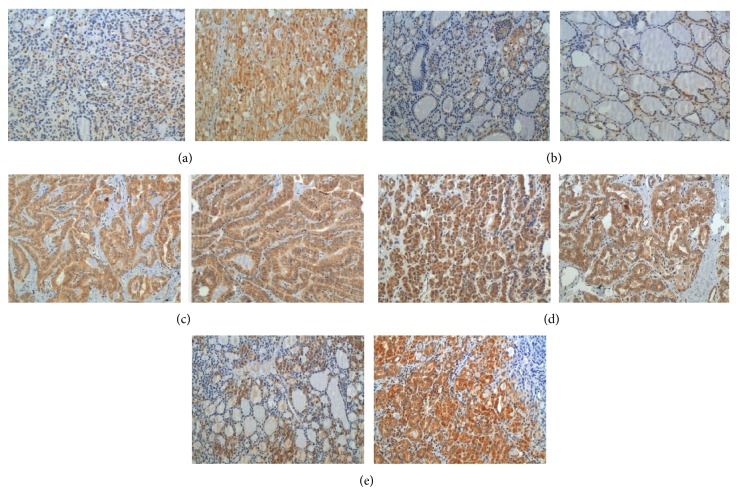
MAGE A3 expression in different lesions, (a) follicular adenoma (×200); (b) goiter (×200); (c) papillary thyroid carcinoma of the classic form (×200); (d) follicular variant of papillary thyroid carcinoma (×200), and (e) follicular carcinoma (×200).

Cells were considered positive for MAGE A3 when a cut-brown staining was observed in the cytoplasm as demonstrated in Figure 2. Visual evaluation was made for each tissue spot, estimating the percentage of positive tumor cells and the staining intensity. The percentage of positive cells was graded as follows: 0 = no positive cell; 1 = up to 25% positive cells; 2 = 25 to 50% positive cells; 3 = 50 to 75% positive cells; 4 = more than 75% positive cells. Intensity was graded as follows: 0 = negative; 1 = faint staining; 2 = moderate staining; 3 = strong staining. A final score was calculated adding both percentage of positive cells and immunostaining intensity, which ranged from 0 to 7. For statistical purposes, cases scored from 0 to 2 were grouped as negative and cases scored from 3 to 7 were considered positive.

In addition to visual evaluation, we also analyzed the immunohistochemical expression of MAGE A3 using the ACIS-III automated cellular imaging system (ChromaVision Medical Systems, Inc, DAKO). Briefly, each tissue spot was digitized to the system software and a numerical value proportional to the intensity and extension of brown staining was attributed by the computer analysis. We considered the given numbers using the following formula: Score = (Intensity × Brown area)/(Brown area + Blue area). The final value of this semiquantitative analysis was given by the mean of triplicates. We considered staining ≤ median as negative, whereas all staining > median was considered positive in the survival analysis, as previously described [14–16].

Regarding tumor-infiltrating lymphocytes analyses were considered positive for immunohistochemical markers when a clear-cut brown staining was observed in the typical corresponding cellular localization. The cases were grouped into categories for statistical analysis: negative (no positive cell) and positive (presence of positive cells in each spot).

### 2.5. Statistical Analysis

The statistical analysis was carried out using the SAS System for Windows (Statistical Analysis System), version 9.1.3, Service Pack 3, Institute Inc., 2002-2003, Cary, NC, USA. A multivariate logistic regression model was applied using tumor type as a dependent variable; protein expression; and clinical risk factors, including gender and age as explicative variables. Recurrence-free survival was calculated using Kaplan-Meier survival curves with log-rank comparison. Nonparametric analysis was performed using either chi-square or Fisher's exact test, as indicated. The Mann-Whitney test was used to compare continuous or arranged measures between two groups; Kruskal-Wallis test was used to compare three or more groups. The predictive accuracy of MAGE A3 expression to predict malignancy was evaluated using receiver operating characteristic (ROC) curve analysis based on predicted probabilities from logistic regression models. All tests were conducted at the significance level *P* = 0.05.

## 3. Results

As expected, DTC patients were predominantly females (81.52%) with a mean age at diagnosis of 45.63 ± 15.34 years (range: 15 to 88 years). DTC group did not differ from the individuals with benign thyroid diseases regarding gender or age at diagnosis. Unfortunately, we did not obtain sufficient metastatic tissues to be included in the statistical analyses; thus, these tissues were excluded from further analysis.

### 3.1. MAGE A3 Evaluation

**Figure 3:**
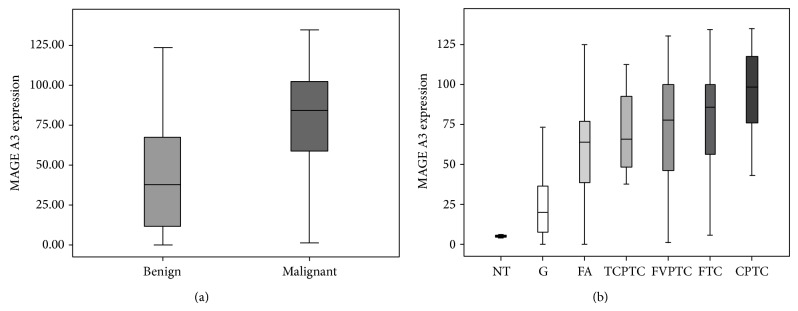
IHC quantitative analysis showing different staining between benign and malignant groups. (a) MAGE A3 immunohistochemical expression in benign and malignant thyroid tissues (*P* < 0.001). (b) MAGE A3 immunohistochemical expression in benign and malignant subtypes of thyroid tissues.

The positivity of MAGE A3 protein was identified in the cytoplasm with a brown color. Intensity and positivity differences were observed among tissue types, both in the quantitative and the semiquantitative analyses, as demonstrated in Figure 3 and Table 1.

According to semiquantitative (visual) analysis, MAGE A3 was positive in 185 (94.87%) out of the 195 cases of DTC and 74 (62.18%) out of the nonmalignant thyroid samples analyzed (*P* < 0.0001). FTCs presented a mean score of 3 for the percentage of positive cells and of 2 for intensity, CPTCs presented a mean score of 3 for the percentage of positive cells and of 2 for intensity, FVPTCs presented a mean score of 3 for the percentage of positive cells and of 2 for intensity, TCPTCs presented a mean score of 3 for the percentage of positive cells and of 3 for intensity, FAs presented a mean score of 2 for the percentage of positive cells and of 2 for intensity, Gs presented a mean score of 1 for the percentage of positive cells and of 1 for intensity, and NTs FAs presented a mean score of 0 for the percentage of positive cells and of 0 for intensity.

A comparison of scores in different subtypes of thyroid lesions revealed that the visual analysis of IHC was able to discriminate some lesions, as demonstrated in Table 1.

ACIS III analysis showed similar results concerning the differential diagnosis of thyroid lesions. MAGE A3 was expressed more in malignant than in benign lesions (Figure 3). Based on the medians of expression given by ACIS III, it was possible to perform a diagnostic ROC curve for thyroid nodules. The ROC curve for a cutoff point of 71.93 may have identified a reasonable point of specificity and sensitivity. In fact, MAGE A3 expression distinguished malignant from benign lesions (*P* < 0.0001) with 66.70% sensitivity, 77.70% specificity, positive predictive value (PPV) of 85.92%, negative predictive value (NPV) of 53.36%, and accuracy of 70.32% (Figure 3). This was also done with the follicular-patterned lesions, which constitute a major pathology challenge for diagnosis: CFT, FA, and FVPTC. The comparison of FTC with FA suggested 78.550 as the cutoff point (FTC group greater than or equal to 78.550 and FA less than that), showing a sensitivity of 71.40%, specificity of 78.80%, PPV of 73.14%, NPV of 77.32%, and accuracy of 75.49%. However, the ROC curve for FVPTC versus FA did not identify a good cutoff point. For a median expression of 76.660 (FVPTC group 76.660 or greater and smaller than that of FA) the sensitivity was 53.30%, the specificity was 75%, PPV was 64.86%, NPV was 64.98%, and accuracy was 64.93% (Figure 3).

Concerning the quantitative analysis, the median expression of MAGE A3 was higher (75.077 ± 30.419) in patients aged >45 years than in patients aged <45 years (85.646 ± 32.625; *P* = 0.0272) and in females (84.763 ± 30.375) than in males (65.840 ± 32.044; *P* = 0.0016). Regarding the characteristics of aggressiveness and invasion, tumor size, and multifocality, MAGE A3 was higher in tumors in which there was extrathyroidal invasion (83.421 ± 30.880) than in cases without invasion (71.765 ± 32.311) (*P* = 0.0206). As expected, MAGE A3 was more expressed in patients with stage II (91.072 ± 22.983) when compared with stage I-TMN (76.060 ± 32.875) (*P* = 0.0107) as demonstrated in Table 2. However, there was correlation neither with tumor size and multifocality nor with disease-free interval.

### 3.2. MAGE A3 Expression and Tumor-Infiltrating Lymphocytes

We observed that MAGE A3 positive tumors were more likely to present CD8+ TIL 50% than CD8− TIL 46% (*P* = 0.0346). When we compared MAGE A3 and CD8 positivity with clinical pathological features, MAGE A3+ CD8+ tumors were associated with less aggressive features: 31% of tumors presenting MAGE A3+ CD8+ phenotype did not present extrathyroidal invasion, whereas only 15% of MAGE A3+ CD8− were not invasive (*P* = 0.0427). In addition, 35% of MAGE A3+ CD8+ tumors were <2 cm, while 13% of MAGE A3+ CD8− were >2 cm (*P* = 0.0034) (Table 3). We did not find any correlation of MAGE A3+ CD8+ with outcome, although there was a trend of these tumors to evolve free of disease (*P* = 0.0634). Our results did not evidence any association between MAGE A3 expression and infiltration of CD4+ TIL.

## 4. Discussion

Many members of the MAGE family are normally expressed in the testis and placenta; however, members of the A subfamily are found mainly in neoplastic tissues, leading some authors to identify the MAGE A family as a candidate of thyroid carcinogenesis mediator [17, 18]. Although there are reports on the expression of members of MAGE family in thyroid cancers, reports concerning MAGE A3 are still insufficient [18–21].

In this work, we demonstrated that MAGE A3 may help not only to identify malignancy in thyroid nodules but also to sort out follicular-patterned lesions (i.e., FTCs and FAs). This was also demonstrated by Ruschenburg et al. for other MAGE family members. The authors showed that the expressions of MAGE A1 and MAGE1/2 detected in fine-needle aspiration biopsies (FNABs) by reverse transcriptase-polymerase chain reaction (RT-PCR) distinguished FVPTC from papillary hyperplasia in nodular goiters, facilitating the diagnosis of thyroid nodules and providing additional information to delineate PTC from papillary hyperplasia in FNAB [21]. Other functional studies reinforced these differences between MAGE expressions in thyroid lesions, suggesting that MAGE A3/6 and FGFR2-IIIb participate in the development of thyroid cancer [22].

We also demonstrated that MAGE A3 expression is related to some features of aggressiveness. Although not exactly similar, other authors have also reported associations between MAGE A3 and clinical pathological features of thyroid tumors. Milkovic et al. demonstrated that MAGE A3 positivity was more frequent in PTCs smaller than 1 cm (microcarcinomas), suggesting that the loss of this protein expression could be related to tumor progression [18]. Using an antibody that recognized both MAGE A6/A3 in 375 cases of thyroid tissues and 53 of metastatic disease, Cheng et al. demonstrated that MAGE proteins are overexpressed in primary and metastatic thyroid tumors when compared with their surrounding normal tissues. Furthermore, cytoplasmic MAGE score was related to tumor size and the number of lymph node metastases [19]. Studying mice models, Liu et al. [23] observed that there was a strong staining of this protein in large tumors and in metastasis, thus, providing additional evidence that this protein may be involved in the formation of metastasis and tumor progression. Using an antibody that only recognized MAGE A, we found no correlation between MAGE A3 expression and metastasis. We observed an increased expression of MAGE A3 in the presence of extrathyroidal invasion, and more advanced stages of disease pointed out to the same direction, leading us to believe that this protein is related to tumor progression and aggressiveness. Furthermore, we demonstrated a possible clinical utility of MAGE A3 expression in the identification and characterization of malignancies of follicular lesions.

Although the real physiologic role of MAGE 3 gene has yet to be identified, the cancer specificity of this gene expression may be important because of its possible application in tumor-specific immunotherapy [4]. In fact, MAGE A3 has been studied as a possible immune target in other types of tumors and may be interesting for differentiated thyroid cancers refractory to radioiodine therapy.

Furthermore, we demonstrated that although MAGE A3 has been related to some features of tumor aggressiveness, when tumors were MAGE A3+ and able to recruit CD8+ expression, aggressiveness not only disappeared but also was associated with better prognostic features such as stage I, smaller tumors (<2 cm), younger age (≤45 years), and absence of extrathyroidal invasion. There was a trend of MAGE A3+ CD8+ tumors to evolve free of disease. These results reinforce the hypothesis of a role of MAGE A3 as a recruiter/inducer of CD8+ tumor infiltrating lymphocytes, a set of immune cells that our group had already associated with better outcome and less aggressive disease [15, 16]. As already mentioned, the members of the MAGE family have been recently considered as tumor antigens, therefore, able to induce and recruit several types of immune cells to attack tumors. In a recent study, Gérard et al. demonstrated that MAGE A3 may be an efficient inducer of long-lasting antitumor responses [10]. In fact, several groups have demonstrated that vaccines using MAGE A3 as immune response inducer might work for different types of advanced cancers, such as lung cancers, melanomas, renal cancers, and esophageal and even head and neck cancers, all with a considerable efficacy [24–27]. There are no reports on the usage of MAGE A3 as an immune vaccine in thyroid cancer until the present moment, but undoubtedly, our study opens this possibility for a promising future scenario, especially considering patients with metastatic or refractory disease.

In conclusion, we demonstrated that although MAGE A3 does not seem to solve diagnostic problems, such as the diagnosis of follicular-patterned lesions, it may play an important role in malignant thyroid nodules, potentially discriminating cases of worse outcome and presenting an interesting perspective for new researches on differentiated thyroid cancer immunotherapy.

## Figures and Tables

**Table 1:** MAGE A3 expression levels according to visual (semiquantitative) IHC in benign and malignant thyroid nodules and in different follicular patterned lesions including classic (CPTC), follicular variant papillary thyroid carcinomas (FVPT), follicular carcinoma (FTC), and follicular adenoma (FA).

Analyzed groups	*P* value (semiquantitative IHC)	Semiquantitative IHC		Predictive value	
		Sensitivity (%)	Specificity (%)	Positive (%)	Negative (%)
Malignant versus benign	<0.0001	94.87	32.35	72.83	76.74
CPTC versus goiter	<0.0001	96.04	49.06	78.86	86.21
CPTC versus FA	0.0215	96.04	15.96	69.29	66.67
CPTC versus variants	N.S.	96.04	0.94	66.90	55.56
FVPTC versus goiter	<0.0001	89.80	49.02	62.86	83.33
FVPTC versus FA	N.S.	89.80	15.69	50.57	61.54
FVPTC versus FTC	N.S.	97.56	10.20	47.62	83.33
FTC versus FA	0.0396	97.56	15.69	48.19	88.89
FTC versus goiter	<0.0001	97.56	49.02	60.61	96.15
FA versus goiter	0.0006	84.31	49.02	62.32	75.76

**Table 2:** Immunohistochemical expression of MAGE A3, according to clinicopathological features of aggressiveness, patient's outcome, and immunological markers.

Clinicopathological features (%)		Quantitative analysis (ACIS) Median	*P* value
Gender	Female (82)	**90.100**	**0.0016**
	Male (18)	72.770	

Ethnicity	White (96)	91.230	0.9069
	Nonwhite (4)	74.480	

Age	≤45 years old (58)	77.055	**0.0272**
	>45 years old (42)	**92.290**	

Tumor size	<2 cm (48)	77.590	0.6526
	2–4 cm (25)	83.065	
	>4 cm (37)	95.510	

Extrathyroidal invasion	Yes (56)	**89.490**	**0.0206**
	No (44)	69.060	

Capsulation	Yes (33)	77.590	0.9570
	No (67)	79.650	

Multifocality	Yes (41)	79.745	0.3918
	No (59)	79.120	

Metastasis at diagnosis	Present (15)	83.065	0.9774
	Absent (85)	79.120	

Disease stage (TNM)	I (63)	**77.325**	**0.0107**
	II (8)	**93.570**	
	III (12)	83.380	
	IV (17)	**92.540**	

Outcome	Free of disease (15)	79.840	0.3475
	Recurrent (85)	98.410	

**Table 3:** Immunohistochemical expression of MAGE A3 and CD8 TIL, according to clinicopathological features of aggressiveness and patient's outcome.

Clinicopathological features	Semiquantitative analysis (visual)		*P* value^*^
	MAGE A3+ CD8+ *N* (%)	MAGE A3+ CD8− *N* (%)	
Age			
≤45 years old	**48 (29%)**	42 (25%)	**0.0422**
>45 years old	28 (17%)	48 (29%)	
Tumor size			
<2 cm	**49 (35%)**	18 (13%)	**0.0034**
>2 cm	36 (25%)	39 (27%)	
Extrathyroidal invasion			
Yes	39 (27%)	38 (27%)	**0.0427**
No	**44 (31%)**	21 (15%)	
Capsulation			
Yes	18 (14%)	23 (17%)	0.0875
No	56 (42%)	35 (27%)	
Multifocality			
Yes	34 (25%)	22 (16%)	0.8585
No	51 (37%)	30 (22%)	
Metastasis at diagnosis			
Present	53 (38%)	39 (28%)	0.2719
Absent	32 (23%)	15 (11%)	
Disease stage (TNM)			
I	**57 (36%)**	27 (18%)	**0.0464**
II	4 (3%)	6 (4%)	
III	8 (5%)	10 (7%)	
IV	17 (13%)	21 (14%)	
Outcome			
Free of disease	68 (48%)	54 (37%)	0.0634
Recurrent	7 (5%)	(10%)	

^*^Fisher's exact test.
